# Predictors of Initial and Sustained Remission in Patients Treated with Antithyroid Drugs for Graves' Hyperthyroidism: The RISG Study

**DOI:** 10.1155/2019/5945178

**Published:** 2019-01-03

**Authors:** J. Karmisholt, S. L. Andersen, I. Bulow-Pedersen, A. Carlé, A. Krejbjerg, B. Nygaard

**Affiliations:** ^1^Dept. of Endocrinology, Aalborg University Hospital, 9000 Aalborg, Denmark; ^2^Dept. of Clinical Institute, Aalborg University, 9100 Aalborg, Denmark; ^3^Dept. of Clinical Biochemistry, Aalborg University Hospital, 9000 Aalborg, Denmark; ^4^Dept. of Oncology, Aalborg University Hospital, 9000 Aalborg, Denmark; ^5^Dept. of Endocrinology and Internal Medicine, Herlev Hospital, 2730 Copenhagen, Denmark

## Abstract

*Purpose. *To study predictors of attaining (part 1) and sustaining (part 2) remission in patients with Graves' hyperthyroidism (GH) treated with antithyroid drugs (ATD).* Methods. *In the prospective first part, the included patients were treated with ATD until a prespecified definition of remission (thyrotropin > 0.4 mU/L and TSH-receptor antibodies (TRAb) ≤ 1. 0 IU/L in a patient receiving a methimazole dose ≤ 5 mg/day, on two occasions two months apart) was met, or for 24 months. In the second part, patients attaining remission in part 1 were randomized to treatment or observation and followed until relapse or for 24 months.* Results. *173 patients completed study 1 and 53% attained remission. TRAb and age were the only significant predictors of remission. Patients with baseline TRAb below vs above 10 IU/L attained remission in 63% compared to 39%, and 5 months priorly (p<0.001). In study 2, 96.4% of the patients randomized to treatment (n=33) sustained remission compared to 66% in the observation group (n=33). Treatment arm was the only significant parameter (p<0.001) of sustained remission.* Conclusion. *Baseline TRAb was prognostic for attaining remission in GH. Consecutive TRAb measurements during treatment were not worthwhile, but a single measurement after 6-8 months in patients with initial TRAb < 10 IU/L could substantially shorten the treatment period in a subgroup of patients. Only 3.6% of the patients in remission experienced relapse during follow-up when treated with a combination of fixed low dose methimazole and L-T4.** ClinTrial.gov registration number is**  NCT00796913.

## 1. Introduction

Thyrotoxicosis including Graves' hyperthyroidism (GH) affects many people worldwide [1–3]. Diagnosing GH is often straightforward, whereas treatment may be challenging. Attaining and sustaining remission is the main goal when treating GH [4, 5]. In most patients, remission is achievable with antithyroid drug (ATD) therapy. However, sustaining a remission after stopping ATD has proven to be more challenging as relapse is seen in around 50% of GH patients after discontinuing ATD [6–9]. Studies, investigating both type and dose of ATD as well as different treatment durations, show that titration therapy with methimazole for 12–18 months is the treatment of choice [9, 10]. This is in line with the recent American and European guidelines on hyperthyroidism [4, 5]. However, ATD treatment also has side effects which may be severe and even fatal [11]. Inducing a hypothyroid state when treating GH may activate or aggravate Graves' orbitopathy and should be avoided [12, 13]. At the disease onset, it is difficult to identify which patients will enter remission during a standard course of ATD treatment. In clinical practice, GH is a disease with highly variable clinical presentation and prognostic outcome. Hence, monitoring treatment with regular measurements of thyroid hormones is necessary to avoid over- or undertreatment. Thus, it is important to optimize the control program during ATD treatment and to identify if there are subgroups of patients who could benefit from a shorter or longer treatment duration compared to the standard treatment period.

The present study comprises two parts and is termed the “Remission Induction and Sustenance in Graves' Disease” (RISG) Study. The first part, termed RISG1 (prospective cohort-study), aimed to investigate how and when individual patients with newly diagnosed GH enter remission during ATD treatment and to study the usefulness of regular TSH-receptor antibodies (TRAb) measurement during treatment. The primary outcome of the second part of the study (RISG2) was to compare, in a randomized approach, the effects of a low-dose block-replace treatment versus no treatment, on keeping patients in remission once remission is achieved.

## 2. Methods

The inclusion and study protocol have been described in detail previously [14]. In brief, newly diagnosed GH patients were recruited from two Danish endocrine referral centers in Aalborg (Aalborg University Hospital) and Copenhagen (Herlev University Hospital) between January 2007 and June 2011. The populations were considered mildly iodine deficient in Aalborg and borderline iodine deficient in Copenhagen [15]. Consecutive patients with TSH < 0.01 mU/L, total T3 above 3.0 nmol/L, and TRAb ≥ 1.0 IU/L or diffuse increased uptake on a thyroid scintigram were invited. Patients were included if the above measurements were confirmed in a subsequent blood sample. Exclusion criteria were age below 18 years, pregnancy or delivery within the previous 12 months, severe comorbidity, imminent or manifest thyroid storm, patients with moderate or severe Graves' orbitopathy considered for immunosuppressive therapy, prior ablative treatment for Graves' disease, treatment with antithyroid medication within 24 months, or known intolerance of methimazole (MMI) or propylthiouracil (PTU). Procedures at inclusion were blood sampling, thyroid ultrasonography, and recording of thyroid related eye signs and symptoms. Furthermore, the patients completed a questionnaire regarding history of thyroid disease, medication including estrogen and nutritional supplements, smoking habits, comorbidities, and presence of thyroid disease in relatives.

The study was approved by the local ethical committee North Denmark Region Committee on Health Research Ethics (VN-20060062) and registered in ClinTrial.gov (NCT00796913). All the patients provided informed consent.

Blood samples were drawn at baseline and after 2-3 weeks, 5-6 weeks, 2 months, 3 months, 4 months, and every second month hereafter until remission or until 24 months after inclusion. The initial ATD dose was MMI 10 mg three times, 10 mg two times, or 15 mg once daily according to whether the initial total T3 level was > 2 or 1.4 – 2 or 1.2 – 1.3 times above the upper reference limit of T3, respectively.

In vivo kinetic studies on ATD have shown dose equivalence between MMI and PTU of 1:15 [16]. In our experience, and hence being applied in the protocol for this study, a dose equivalence of 1:20 has proven to be clinically preferable. So, an equivalent dose of 5 mg MMI was 100 mg PTU. We report PTU doses in MMI dose-equivalents.

**Figure 1:**
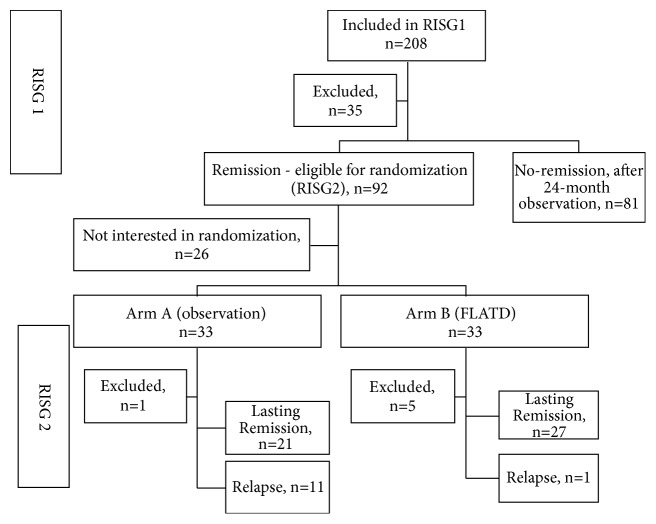
Flow chart of the inclusion process comprising the two substudies RISG1 (predictor of attaining remission) and RISG2 (comparing relapse rates in patients randomized to observation or fixed low dose antithyroid drug treatment; FLATD).

Remission was defined as TSH > 0.4 mU/L and TRAb ≤ 1. 0 IU/L in a patient receiving a methimazole dose ≤ 5 mg/day, on two occasions two months apart. The patients who entered remission were subsequently invited to participate in the randomized open-label study (termed RISG2), in which the patients were randomized to either continuous fixed low-dose antithyroid drug treatment (FLATD) or just observation. FLATD was MMI 5 mg/day in combination with L-T4 of 1 microgram/bodyweight in kilogram/day. Dose adjustments were only made on the L-T4 dose, in order to maintain normal TSH. In this part of the study, the patients were followed until relapse or for 24 months. Relapse was defined as TSH < 0.01 mU/L and total T3 ≥ 3.0 nmol/L and TRAb ≥ 1.0 IU/L. The patients were investigated at randomization and after completing the study and had regular blood samples taken at the time of randomization and after 1, 3, 6, 9, 12, 18, and 24 months. At the investigations the patients underwent the same procedures as performed at baseline.  Figure 1 shows an overview of the inclusion from the prospective observation study (RISG1) until the end of the randomized study (RISG2). During the study, the patients were seen at routine clinical controls and were contacted regularly in order to preserve compliance with the randomization arm procedure and blood samplings and for registration of possible adverse events.

## 3. Assays

At the time of the study, the clinical routine at the two centers was to use total T4 and total T3 levels for diagnosing and treating thyroid disease, with a single measurement of thyroxine-binding globulin (TBG) or a T3-test to evaluate thyroid hormone binding capacity. Serum total T4 concentration (reference range: 60–140 nmol/L, in both involved laboratories), serum total T3 (reference range: Aalborg 1.1–2.5 nmol/L, Copenhagen 1.0–2.6 nmol/L), and serum TSH (reference range: Aalborg 0.30–4.5mU/L, Copenhagen 0.40–4.0 mU/L) were measured by standard high capacity analytical platforms (Aalborg, Roche Diagnostics Elecsys; Copenhagen, Immulite 2500, chemiluminescent enzyme immunoassay). TRAb was measured using a receptor assay (DYNOtest TRAK, Thermo Fisher, Berlin, Germany). TRAb ≥ 1.0 IU/L was considered TRAb positive [17]. TPO-Ab detection limit was 30 kU/L by KRYPTOR antibody tests (Thermo Fisher, Berlin, Germany) [17]. Urinary iodine was determined by the Ceri/Arsenium method after alkaline ashing [18, 19] and expressed as crude concentrations (*μ*g/L).

### 3.1. Statistical Analysis

Data were analyzed using IBM SPSS statistics version 24.0 (Armonk, New York, USA). Mann-Whitney* U *and Wilcoxon signed rank (nonparametric) tests were used for comparisons between groups and within groups on numeric data. Chi square or Fisher's exact test was used on categorical data. The log rank test was used to test for differences on the Kaplan-Meyer survival analysis. Multiple logistic regression analyses were performed with remission as dependent categorical variable and the significant variables in Table 1 as independent variables. Area under curve (AUC) calculations were used for studying differences on the receiver operating characteristic (ROC) curves at different TRAb cut-off levels (TRAb equal to or lower than 5, 7, 10, 15, or 20 IU/L) and age cut-off levels (age equal to or lower than 40, 45, 50, or 55 years). A p value < 0.05 was considered as statistically significant.

## 4. Results

A total of 208 patients were included. Table 1 shows the patients' characteristics and the results of the thyroid function tests and antibody levels at baseline for the 173 (83.2%) patients who completed the first part of the study protocol (RISG1) at the two centers. Most patients were recruited in Aalborg (80%) with comparable completion rates of 83 % at the two centers. The reasons for not completing RISG1 were noncompliance (15 patients), development of severe orbitopathy (7 patients), moving out of the area (4 patients), side effects of MMI/PTU (4 patients), development of major comorbidity (3 patients), hemithyroidectomy due to follicular neoplasia (1 patient), and pregnancy (1 patient). Median (IQR) age, TRAb level, and T4 level at baseline for the excluded patients were 48 (31–54) years, 12 (5.8–16.8) IU/L, and 202 (179–233) nmol/L, respectively. One patient was TRAb negative and one had a missing TRAb value at baseline.

Twenty-nine patients (17%) in RISG1 and 11 (17%) in RISG2 used oral contraceptives or estrogen supplements. The median (interquartile range, IQR) of the thyroid hormone levels at baseline in these patients was as follows: total T4 235 (204–312) nmol/L, total T3 5.8 (4.4–8.2) nmol/L, compared to total T4 205 (171–256) nmol/L, total T3 5.4 (4.0–7.0) in nonestrogen users. The median (interquartile range, IQR) level of TBG was 26.0 (20.5–31.5) mg/L in the estrogens user and 18.0 (16.0–22.0) mg/L in the nonestrogen users. Patients using oral contraceptives or estrogen supplements were excluded from the statistical analyses when total T4 or total T3 was included.

Ninety-two patients entered remission during RISG1 (Figure 1) and willing patients (n = 66) were subsequently randomized to either observation without treatment (n= 33, Arm A) or FLATD (n= 33, Arm B). The patients' characteristics at the time of randomization are seen in Table 2. One patient in arm A was excluded due to withdrawal of consent, whereas 5 patients were excluded in arm B (1 due to withdrawal of consent, 1 due to inability to follow the blood-sample protocol, 1 due to development of joint pain, 1 due to pregnancy, and 1 death (due to metastasizing cholangiocarcinoma diagnosed 3 months after randomization)). Regarding adverse events, none were registered in the observation group. Two patients (6%) were registered with adverse events in the treatment group, one patient developed joint pain and was excluded from the study, and one had a skin rash, which disappeared when the L-T4 tablet was switched to another brand.

### 4.1. Predictors of Remission (RISG1)

The median (IQR) observation time was 22 (12-24) months and 92 patients (53.2 %) entered remission after a median (IQR) of 13 (10–17.75) months. The patients entering remission were comparable to the nonremission patients on all parameters (Table 1) except they had lower concentrations of TRAb and lower T4 levels, were younger, and had higher BMI, at baseline. The significant parameters were entered as independent variables in a multivariate logistical regression model with remission as dependent variable. In this model, only low TRAb (p= 0.005) and younger age (p=0.013) were associated with remission as an outcome, with odds ratios 1.047 (95% CI: 1.014–1.081) and 1.033 (95% CI: 1.007–1.060), per unit, respectively. Thus, a 35-year-old patient with initial TRAb of 5 IU/L had an odds ratio of entering remission of 1.6 compared to a 55-year-old with initial TRAb of 10 IU/L.

**Figure 2:**
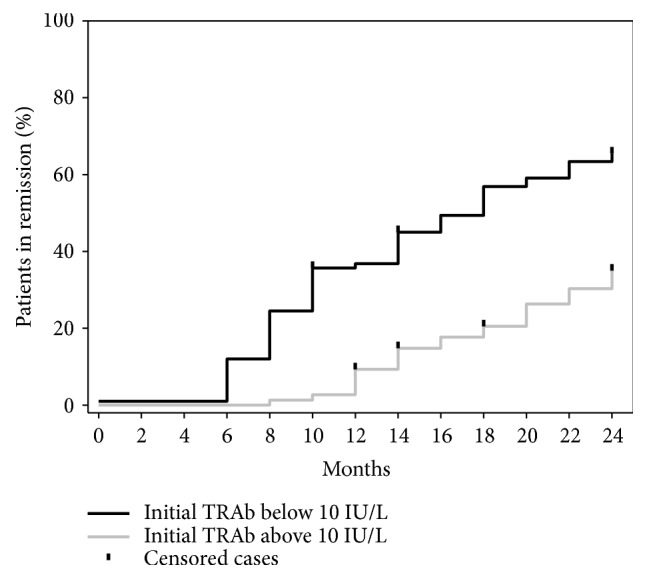
Event curve of patients achieving remission depending on whether initial TRAb was below (black) or above (grey) 10 IU/L. Remission was TRAb < 1.0 IU/L and TSH ≥ 0.40 mU/L on a MMI dose of 5 mg or less per day evaluated twice, two months apart.

In order to examine which TRAb and age value were the strongest predictor of remission, several ROC curves and corresponding AUC values were calculated (data not shown). The cut-off point of TRAb at 10 IU/L showed the highest AUC (0.652), but still the sensitivity and specificity were rather low at 0.57 and 0.74, respectively. An event curve for entering remission depending on whether the baseline TRAb value was below or above this cut-off of 10 IU/L is shown in Figure 2. After 24 months of observation, 63.3% (n=62) of the patients with a baseline TRAb value below 10 IU/L entered remission, whereas only 39.4% (n=28) with a baseline TRAb value above 10 IU/L entered remission. The mean time of entering remission was 16.4 vs. 21.5 months (log rank, p=<0.001) in patients with TRAb below vs. above 10 IU/L, respectively. TRAb was significantly lower at 6.6 (4.0–12) vs. 10 (7.2–18) IU/L (p<0.001) among the 37.6% of patients who had one or more elevated TSH measurements (indicator of overtreatment) during the study. Receiving 5 mg MMI or less per day was a prerequisite in the definition of remission. We thus calculated which of the 16 consecutive TRAb measurements were most informative in patients receiving 5 mg MMI or less per day, in order to identify patients in remission. In such patients, TRAb measurements at 6 or 8 months identified remission in 48% and 46%, respectively. When subdividing such patients according to initial TRAb level, TRAb measurements at 6 months identified remission in 56% in patients with initial TRAB below 10 and in 13% with initial TRAB above 10 IU/L. At time points other than 6 or 8 months, a TRAb measurement only identified remission in around 20%, with no major difference between patients with initial TRAb below or above 10 IU/L.

### 4.2. Risk of Relapse (RISG2)

**Figure 3:**
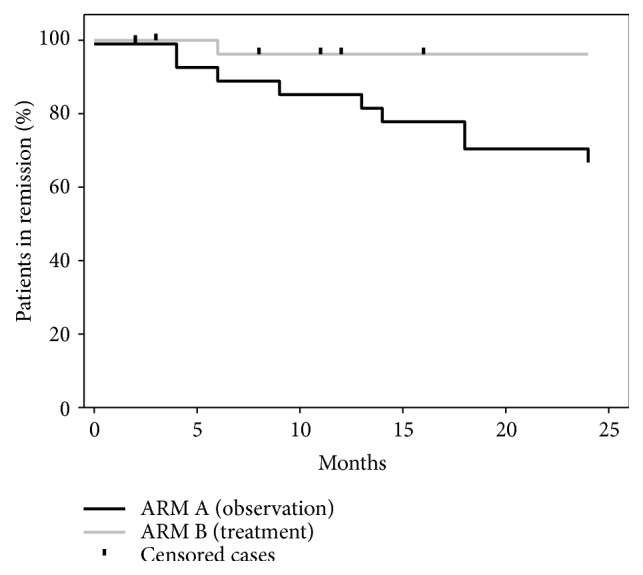
Kaplan-Meyer curve of relapse of Graves' hyperthyroidism in patients randomized to either fixed low dose block-replace treatment (grey) or just observation (black).

Significantly more patients completing the study had relapse in the group randomized to observation as 11 out of 32 (34.4 %) patients had relapse in this group compared to only 1 out of 28 (3.6%) in the group randomized to treatment (Chi^2^, p < 0.003).  Figure 3 shows the event curve of risk of relapse in the two arms. The mean time to relapse was significantly longer in the treatment arm (observation, arm A, 20.1 (17.4–22.8); treatment, arm B, 23.3 (22.0–24.6) months, log rank p= 0.003). None of the parameters in Table 2, TRAb levels at the time of diagnosis or time to achieve remission in RISG1 were significantly different between the patients who experienced relapse and patients in lasting remission. After the randomization, thyroid volume was coincidently different between the two groups (Table 2). In a multivariate logistic model (relapse as dependent variable, thyroid volume and treatment arm as independent variables) treatment arm (p < 0.003; odds ratio, 13 (95 % CI: 1.5–108)) was the only significant parameter. When only patients completing the study (per protocol analysis) were studied, the same results were found (data not shown).

## 5. Discussion

TRAb is a hallmark of GH. It is a diagnostic marker of the disease [20], a prognostic marker of relapse after remission [7, 21], and a risk marker of developing Graves' orbitopathy [22]. With the present study we confirmed that TRAb was a prognostic marker and showed that patients with lower TRAb were at higher risk of overtreatment (elevated TSH). However, it was not possible to make more advanced models to better predict remission, by including further parameters including smoking, GD in relatives, or a close follow-up by multiple TRAb measurements. Different predictive scoring systems were proposed for calculation of risk of relapse in Graves' disease after antithyroid treatment [23–25]. When the individual components of these scores were evaluated thyroid volume and fT4 level were the significant parameters in the Italian Clinical Severity Score [25]. In the Dutch GREAT score, younger age, high fT4, higher TRAb, and presence of goiter were the significant parameters [23]. In an external validation study of the GREAT score, performed by Struja et al., only higher TRAB value was significant in the multivariate analysis [24]. The AUC in these studies were between 0.60 and 0.67 and comparable to 0.65 as found in the present study, where only a simple stratification on TRAb below or above 10 mU/L was used.

The generally used definition of remission in GH is “the patients has not experienced relapse of Graves' disease 12 months after stopping ATD” [4]. This definition is retrospective and is thus difficult to apply into clinical use when treating patients with GH, as the clinician would prefer to ascertain remission before treatment is withdrawn. Using a definition of remission founded on measurable parameters could enable shorter treatment duration based on individual response to treatment. We used a definition of remission as TRAb < 1.0 IU/L and TSH ≥ 0.40 mU/L on a MMI dose of 5 mg or less per day evaluated twice, two months apart. In patients randomized to 24 months of observation after entering remission, 66% were still in remission after 2 years of observation. The remission rate found in other prospective studies on patients with GD treated with ATD was highly variable, ranging from 20 to 70% [23, 26–39]. The majority of these studies showed remission rates between 40 and 70 %, but quite diverse definitions of remission were used. TRAb is probably a relevant element in a definition of current remission in GH. Measuring TRAb every second month as performed in this study is presumably not cost efficient. However, we showed that, in patients with initial TRAb below 10 IU/L, approximately 50% would be in remission (according to the definition above) after 6 to 8 months if the patient had normal TSH on a MMI dose of 5 mg or less per day for the previous two months. Patients in remission according to this criterion randomized to either observation or continuous low dose ATD treatment experienced relapse in 34% or 3.6% of the cases during a 24-month follow-up period. The continuous low dose ATD combined with L-T4 treatment option was well tolerated and with significant lower relapse rate than just observation and could thus be a worthwhile option in patients where a relapse rate of 34% is unacceptable.

The study had some limitations. We used total thyroid hormone measurements as opposed to free T4 and free T3 measurements. However, we measured protein-binding capacity in all patients and as different assays were used in the two centers, treatment and diagnostic cut-off were according to magnitudes of the reference limits of the particular assays, thus increasing the external validity of the results. Free thyroid hormone immunoassays are not without analytical pitfalls and show considerable between-assay differences [40, 41]. Also, most of the conclusions of the study are based on TSH and TRAb measurement, while T4 and T3 values were secondary parameters. In order to further identify relevant risk factors for remission or relapse a more comprehensive clinical work-up and the use of genetic markers could have been valuable [23]. Although the second part of the study was randomized, it was not blinded. The lack of blinding introduces the risk of judging potential adverse effects skewed. Overall, the adverse event rate was very low and the lack of blinding probably had an only negligible effect on the conclusions.

This study addresses several relevant issues when treating GH with ATD. Based on the results from the present study a rational approach to thyroid function testing (TFT, meaning TSH and T3 or T4) and TRAb measurements in patient treated with ATD for GH could be as follows: at the time of diagnosis: measure TFT and TRAb. At this time point TRAb is a diagnostic marker and a TRAb level below 10 IU/L is a risk marker of overtreatment and a favorable marker of attaining remission. Measurement of TFT every 4-6 weeks for the first 4 months and every second to third months hereafter is sensible. Patients with baseline TRAb below 10 IU/L may well have a TRAb measurement performed at the time point 6-8 months if TSH is normal on a MMI equivalent dose of ≤ 5 mg/day. Around 50% of such patients would have a TRAb level below 1.0 IU/L and a likely relapse rate of 34% for the following 24 months if treatment is stopped even after this short treatment duration.

In conclusion, this study showed that TRAb measured at the time of diagnosing GH was a prognostic marker of attaining remission of the disease, but also a risk marker for overtreatment. Consecutive TRAb measurements during treatment were not worthwhile, but a single measurement of TRAb after 6-8 months in patients with initial TRAb < 10 IU/L who have normal TSH on a daily MMI dose of 5 mg or less per day could substantially shorten the treatment period in around 50% of these patients. Lastly, we showed that, in patients in remission, only 3.6% experienced relapse during 24 months of follow-up when treated with a fixed dose of 5 mg MMI in combination with L-T4 supplement. A relapse rate significantly lower than the 34% was seen in the patients randomized to no treatment.

## Figures and Tables

**Table 1:** Baseline characteristics of the included patients. Remission was prespecified and defined as TRAb < 1.0 IU/L and TSH ≥ 0.40 mU/L in a patient on a MMI dose of 5 mg or less per day on two blood samples taken two months apart.

	Remission	No remission	All	P (remission vs no remission)^ #^
Participant, n (%)	92 (53.8)	81 (46.2)	173 (100)	-
Sex, M/F (%F)	13/79 (85.9)	10/71 (87.7)	23/150 (86.7)	0.824
Age, years, median (IQR)	43 (34 – 51)	48 (39 – 55)	44 (36.0 – 52.5)	**0.024**
History of hyperthyroidism, n (%)	4 (4.3)	5 (6.3)	9 (5.2)	0.736
Smoker, n (%)	26 (28.0)	22 (27.5)	48 (27.7)	0.867
Estrogen use, n (%)	15 (16.1)	14 (17.5)	29 (16.8)	1.00
GD in 1st generation relatives, n (%)	22 (23.7)	22 (27.5)	44 (25.4)	0.381
BMI kg/m^2^, median (IQR)	23.5 (21.1 – 27.1)	22.5 (20.1 – 25.6)	23.0 (20.7 – 26.1)	**0.034**
None or minor eye symptoms, n (%)	73 (78.5)	53 (62.5)	127 (73.4)	0.172
Thyroid volume ml, median (IQR)	19.7 (13.2 – 26.7)	20.5 (15.0 – 35.8)	20.0 (14.6 – 30.9)	0.187
S-T4 nmol/L, median (IQR)	203 (165 – 250)	227 (187 – 285)	211 (175 – 263)	**0.010**
S-T3 nmol/L, median (IQR)	5.4 (4.0 – 6.8)	5.5 (4.6 – 8.2)	5.4 (4.1 – 7.2)	0.139
S-TRAb IU/L, median (IQR)	7.00 (3.80 – 11.1)	11.5 (7.73 – 22.0)	8.9 (5.1 – 15.6)	**<0.001**
S-TPO kU/L, median (IQR)	206 (30 – 1791)	459 (30 – 3600)	268 (30 – 2645)	0.443
s-TG ab	35 (16 – 104)	25 (10 – 147)	28.5 (14 – 110)	0.406
u-iodine, *µ*g/L, median (IQR)	132 (80- 208.5)	159 (98.3 – 284.8)	141 (86 – 264)	0.144

^#  ^Mann-Whitney or Chi square/Fisher exact test as appropriate.

n = number.

M = male.

F = female.

IQR = interquartile range.

**Table 2:** Patient's characteristics at time of randomization to either Arma A (observation) or Arm B (treatment with fixed low dose block-replace treatment, FLATD).

	ARMA A (Observation)	ARMA B (FLATD)	P value (Arm A vs. Arm B)^#^
Participant, n	33	33	-
Sex, M/F (%F)	3/30 (90.9)	9/24 (72.7)	0.108
Age, years, median IQR	43 (37 – 53)	46 (36 – 53)	0.649
History of hyperthyroidism, n (%)	2 (7.1)	1 (3.3)	0.605
Smoker, n (%)	9 (27.3)	7 (21.2)	0.775
Estrogen use, n (%)	5 (15.2)	6 (18.2)	0.559
GD in 1st generation relatives, n (%)	9 (27.3)	7 (21.2)	0.775
BMI kg/m^2^, median (IQR)	26.0 (22.9 – 28.9)	27.4 (24.3 – 32.0)	0.184
None or minor eye symptoms, n (%)	24 (72.7)	27 (81.8)	0.488
Time to remission in RISG1, months	13.7 (5.3)	13.6 (5.4)	0.945
Thyroid volume ml, median (IQR)	24 (16 – 32)	17 (12 – 24)	**0.022**
S-T4 nmol/L, median (IQR)	97 (83.5 – 111.5)	104 (87.5 – 112)	0.568
S-T3 nmol/L, median (IQR)	1.7 (1.4 – 1.8)	1.7 (1.4 – 2.0)	0.350
S-TRAb IU/L, median (IQR)	0.5 (0.3 – 0.75)	0.6 (0.3 – 0.8)	0.413
u-iodine, *µ*g/L, median (IQR)	74.5 (45.5 – 133)	74 (41.5 – 103)	0.455

^#  ^Mann-Whitney or Chi square/Fisher exact test as appropriate.

n = number.

M = male.

F = female.

IQR = interquartile range.

## Data Availability

Data supporting the results are stored at the region of North Jutlands (Denmark) platform for clinical and scientific data. The data are accessible for the authors and a limited number of key personnel. On request, relevant data can be provided at any time.
